# Risk prediction tools in multiple long-term conditions management: a qualitative study

**DOI:** 10.3399/BJGP.2025.0371

**Published:** 2026-03-24

**Authors:** Stella Arakelyan, Atul Anand, Stewart W Mercer, Clare Elizabeth MacRae, Nazir Lone, Bruce Guthrie

**Affiliations:** 1 Advanced Care Research Centre, Usher Institute, University of Edinburgh, Edinburgh, UK; 2 Centre for Cardiovascular Science, Chancellor’s Building, University of Edinburgh, Edinburgh, UK; 3 NHS Lothian, Royal Infirmary of Edinburgh, Edinburgh, UK

**Keywords:** decision support techniques, multimorbidity, multiple long-term conditions, patient-centred care, risk prediction tool, risk stratification

## Abstract

**Background:**

Risk stratification is integral to providing good care, but its value in managing patients with multiple long-term conditions (MLTC) remains uncertain.

**Aim:**

To explore the perspectives of healthcare professionals, patients, and carers on the benefits and the challenges of using risk prediction in MLTC management.

**Design and setting:**

Analysis of interviews with 30 professionals and six focus groups with 28 patients with MLTC/carers in four Scottish integrated health and social care partnerships was undertaken.

**Method:**

Data were collected between May 2023 and May 2024, and analysed thematically.

**Results:**

Three themes were identified: legitimation of risk prediction tools, workload implications of risk prediction, and reconfiguration of risk prediction tools. Healthcare professionals questioned the clinical utility of existing tools, noting a lack of clinical nuance and overreliance on blunt algorithms. They stressed increased workload implications of new tools and the need for seamless integration, clearer guidance on how to respond to prediction, and inclusion of psychosocial factors and meaningful outcomes. Artificial intelligence (AI) and routine data were seen as promising in enhancing predictive accuracy and real-time application of new tools, but effective use requires better IT systems, training, and clinical oversight — ‘human-in-the-loop’. Patients/carers expressed mixed views, warning that risk communication could cause unnecessary anxiety, undermine autonomy, and be less relevant in older age. They emphasised the importance of risk communication that reflects social context and aligns with patient priorities.

**Conclusion:**

There was some support for using AI-informed tools for risk stratification, provided they have no workload implications, complement clinical judgement, and account for patient clinical complexity and preferences.

## How this fits in

There are a multitude of risk prediction tools created by researchers, but most are never implemented in practice or rarely used by clinicians. New methods including artificial intelligence (AI) promise ‘better’ prediction tools, but they will not improve care if they are not implemented or used. Participants identified increased workload, poor integration within clinical workflow, unclear guidance on how to respond to prediction tool output, and risk of causing unnecessary anxiety to patients as key barriers to clinical use. AI and routine data offer promise for real-time risk prediction but need strong IT systems, training, and clinical oversight in development and implementation to be effective.

## Introduction

Multiple long-term conditions (MLTC) are increasingly prevalent, driving increased demand for health and social care.^1,2^ In many countries, acute hospital services and primary care are challenged by a steady increase in unplanned care required by patients with MLTC and complex needs.^3^ Proactive, personalised, multidisciplinary care for high-risk patients who use services frequently is commonly proposed as one way to delay and/or reduce future care needs, and in particular to minimise future unplanned care.^4^ Proactive care assumes ‘case finding’ to target higher-intensity support. Case finding is commonly supported by risk prediction models or tools that use multivariable algorithms to stratify individuals’ risks for adverse outcomes (such as falls, fractures, unplanned care, delirium, sepsis, mortality) based on their characteristics, clinical signs, medication regimen, and laboratory tests (Patients at Risk of Re-hospitalisation, Risk Stratification Index 3.0).^5,6^ Risk communication to high-risk patients may encourage them to partake in joint care decision making and advanced care planning, and may increase motivation for therapy adherence and behavioural risk modifications.^7,8^ Risk stratification, therefore, has the potential to allow efficient targeting of scarce healthcare resources to avoid overtreatment of low-risk and undertreatment of high-risk patients.^9^ Clearly, any risk stratification tool has to be shown to accurately predict its chosen outcomes to ensure its use in routine practice,^10–12^ but there are multiple other factors affecting effective adoption (such as time demands, difficulty selecting the most suitable tool).^13,14^

As MLTC prevalence rises,^15^ more person-centred and targeted care is needed. The UK National Institute for Health and Care Excellence multimorbidity guidelines call for better identification of high-risk patients who would benefit most from tailored approaches.^16^ Risk stratification is integral to providing safe and effective care, but its value in managing patients with MLTC remains uncertain. This study aimed to explore the perspectives of health and social care professionals, patients, and carers on the potential benefits and problems of using risk prediction in MLTC management.

## Method

### Setting, design, and participants

This study is part of the Artificial Intelligence and Multimorbidity: Clustering in Individuals, Space and Clinical Context (AIM-CISC) programme,^17^ and examines how prediction tools could be best deployed in clinical practice. AIM-CISC involved a public reference group (six public contributors, including patients with MLTC, carers, professionals, service commissioners) throughout the entire programme to support the research aims. Their input informed the framing of the study, guided our approach to exploring perceptions of using risk prediction in clinical practice, and contributed to the interpretation and contextualisation of key findings.

The design was a qualitative study using interviews and focus group discussions with multidisciplinary healthcare professionals, patients with MLTC, and carers. The setting was a large Scottish health board, and its four constituent integrated health and social care partnerships (HSCPs). Four HSCPs (one city and three mixed urban/rural areas) are collectively responsible for planning and delivery of health and social care services to a population of approximately 900 000 people — around 17% of Scotland’s total population.

We used a purposive sampling approach with snowballing to identify and recruit health and social care professionals working in both community and hospital settings. Eligible professionals were those involved in the delivery of care and/or planning and managing care transitions for patients with MLTC. The research team members (the first, second, and third authors) made initial contact with eligible healthcare professionals electronically, inviting them to take part in the study. Social care professionals were recruited through Enabling Research in Care Homes (ENRICH) Scotland.^18^ Following the interviews, professionals were asked to nominate further potential participants, with sampling for heterogeneity of settings (health/social care, community/hospital care) and disciplines (medical, nursing, allied health professional, social care).

In collaboration with the NHS Research Scotland primary care network, we used a purposive sampling approach to identify and recruit patients with MLTC and carers to participate in focus group discussions. Eligible patients were those aged ≥40 years, living with ≥2 long-term conditions, being on ≥7 prescribed medications, and having recent experience in health and social care, or were carers for such a person. The age cut-off was set at ≥40 years to capture patients with early onset of MLTC who experience clinically significant treatment burden and complexity, and for whom risk prediction tools could meaningfully support clinical decision making and care planning. Patients were sampled from six GP practices recruited by the primary care network and invited by their GP practice to contact the research team for more information if they were interested in taking part.

### Research team and data collection

The research team comprised a health services researcher, primary care researchers, and clinical academics. One health services researcher (the first author) conducted semi-structured interviews with health and social care professionals either remotely (MS Teams videoconference) or face to face (in GP practice or hospital) between May 2023 and March 2024. The same researcher held six focus groups with patients and carers recruited across six GP practices between February and May 2024. The interviews lasted between 40 and 75 min, and the focus group discussions between 95 and 120 min. Topic guides for the broader study examining data-driven clustering of MLTC and practical ways for optimising care for patients with MLTC using risk prediction tools were developed by two authors (the first author and the senior author) experienced in health services research and qualitative methods. Box 1 lists key questions underlying the analysis for this study. The broader interview guide was piloted with two healthcare providers to check comprehension. The focus group guide was piloted in a group discussion with five patients with MLTC/carers who were drawn from the public contributors’ reference group supporting the wider (AIM-CISC) programme.^17^ During all focus groups, the same set of core questions was discussed. The recruitment, data collection, and analysis occurred iteratively, and we continued recruiting until we reached a point of thematic saturation.^19^

Box 1. **A list of discussion questions**

**Healthcare professional interviews**

- Which patients with multiple long-term conditions are at the most risk of adverse events or things going wrong for them? How do they get identified at the moment?
- Do you use any prediction tools in your care at the moment (prompt for, for example, falls risk, pressure ulcer risk, cardiovascular disease prediction)?
- Why do you use or do not use such prediction tools?
- What are the barriers and facilitators to use?
- Would a prediction tool for identifying patients with multiple long-term conditions at high risk of adverse events potentially be helpful to you?
- What key adverse events would you be interested in knowing the risk for?
- What would make the new prediction tool more usable?

**Patient and carer group discussions**

Healthcare professionals often use calculators called risk prediction tools to predict a patient’s risk of undesirable health events. For example, it is common to calculate someone’s risk of future heart disease to decide on whether to start them on a statin drug to lower cholesterol. Another example is that Scottish GPs are asked to review patients at high risk of emergency admission to hospital to see if there is anything they can do to prevent an admission.

- Thinking about what matters to you in particular, what do you think is the most important thing to try to prevent? (prompt for, for example, falls, hospital admission, heart attack, stroke, or moving into a care home).
- How do you feel about healthcare professionals calculating your risk of things like [… as identified by patients/carers] in this way?

### Ethical considerations

The study was approved by the North West — Greater Manchester West Research Ethics Committee (REC reference: 22/NW/0313) and the NHS Health Board. All participants were sent information about the study before the interview and offered the opportunity to ask questions. Eligible health and social care professionals returned signed consent forms prior to interviews. Written consent was obtained from all patient and carer participants immediately before the group discussions. Consent included permission for audio-recording and transcription, and the use of direct quotations in publications, ensuring confidentiality, anonymity, and the right to withdraw from the study at any time.

### Analysis

The interview and focus groups were audio-recorded using an encrypted recorder, transcribed, and imported to NVivo v12 software (QSR International Pty Ltd, 2018) for inductive thematic analysis.^20^ We used inductive thematic analysis to remain grounded in participants’ accounts and capture the diverse perspectives of professionals, patients with MLTC, and carers on the potential benefits and challenges of using risk prediction in MLTC management. After familiarisation with all transcripts and fieldnotes taken immediately after the interviews and focus groups, one researcher (the first author) coded 10 transcripts. The initial coding structure was discussed with a senior researcher (the senior author) and peer-debriefed with a research team. Emerging themes were discussed, and a final coding framework was developed and agreed over several meetings between the team members. The final coding framework was applied to all the transcripts. The codes were aggregated into categories and overarching themes. Themes were evaluated critically by constantly comparing coded data within and across themes to identify emerging patterns and associations.^20^

The research team used several strategies to enhance the trustworthiness of the findings.^21^ To ensure transparency in analysis and reporting, we used processes allowing for an audit trail of data collection, management, and analysis. Two professionals cross-verified the transcripts to ensure accurate representation of interview content. Findings are reported following the Standards for Reporting Qualitative Research guidance.^22^

## Results

Thirty healthcare professionals (22 [73.3%] of whom were women) participated. Most professionals had >15 years of professional experience (range 6–39 years) and had been in their current roles on average for eight years (range 1–25 years). Eight participants held managerial positions and six occupied senior clinical roles in community and hospital settings (Table 1).

Twenty patients with MLTC (nine men), six carers (five women), and two patients with MLTC who also had carer roles (women) participated in six focus group discussions. Over half of patients/carers were aged >70 years (mean 70 years, range 51–89 years) and were cohabiting with another person (Table 2).

Three overarching themes were developed:

- legitimation of risk prediction tools (clinical credibility, practical acceptability, usefulness);
- workload implications of risk prediction; and
- reconfiguration of risk prediction tools.

**Table 1.** Healthcare professional characteristics

Characteristic	Total *n*
**Age, years**	
20–29	2
30–39	10
40–49	8
50–59	10
**Gender**	
Women	22
Men	8
**Setting**	
Community (primary care, community clinics, HSCPs)	18
Hospital	12
**Professional role**	
Older people’s community service manager	2
Clinical pharmacy service manager	1
Care home manager or deputy manager	3
Community link worker or service manager	2
Hospital clinical nurse manager	1
Consultant geriatrician	2
General medicine or acute care consultant	3
Old age psychiatrist	1
Hospital advanced nurse practitioner	2
GP	4
Out-of-hours GGP	1
Allied health professional^a^	4
Clinical pharmacist	2
Social worker	2
**Years of experience since qualified**	
<5	—
5–9	3
10–14	5
≥15	22
**Years of experience in current role**	
<5	12
5–9	11
10–14	2
≥15	5

^a^Physiotherapist *n* = 2, occupational therapist *n* = 2. HSCP = health and social care partnership.

**Table 2.** Patient and carer characteristics

Characteristic	Total *n*
**Age, years**	
50–59	8
60–69	4
70–79	14
80–89	2
**Gender**	
Women	18
Men	10
**Ethnicity**	
Scottish	23
Other British	5
**Region**	
Midlothian	7
East Lothian	5
Edinburgh	16
**Status**	
Patient with MLTC	20
Informal carer	6
Both	2
**Living arrangements**	
Live at home with another person	17
Live at home on my own	10
Live in sheltered (retirement) housing on my own	1

MLTC = multiple long-term conditions.

### Legitimation of risk prediction tools

Health and social care professionals identified older people with frailty and/or cognitive or visual impairments, people with both mental and physical conditions, and the less affluent as being at higher risks of adverse events. These patients were perceived as ‘less predictable’*,* in part owing to more variation in self-management and treatment adherence. Professionals noted the absence of proactive monitoring systems to detect early signs of deterioration in these patients and emphasised the need for tailored, intensive support to mitigate health risks in this population. Patients and carers identified stroke, heart attack, falls, sepsis, seizures, mental health, mobility decline, and emergency hospital admissions as adverse events where early prevention was important.

GPs viewed risk prediction tools as useful for informing clinical decisions for people with a single condition, with commonly used tools including ASSIGN (cardiovascular risk),^23^ Wells score (deep vein thrombosis risk),^24^ CHA2DS2-VASc (stroke risk in atrial fibrillation),^25^ HAS-BLED (bleeding risk on anticoagulation),^26^ and rules-based tools for potentially inappropriate prescribing such as the Scottish Therapeutic Utility^27^and STOPP-START.^28^ However, for people with MLTC at risk of adverse events, identification was predominantly done opportunistically at the point of service use:

*‘I don’t use any of these tools. For all patients who would fit into the MLTC or frail category, I'm assessing that by looking at how well-kept they are and how they walk into the room. Have they become breathless? Do they need a stick? Are they able to give me a history? … All that goes into my assessment, and it’s not a formal thing but experiential.’* (Community GP, 8 years’ experience)

For this population, there was concern that prediction tools were less reliable than in ‘simpler’ patients:

*‘I have more belief in tools in patients with less complexity … What my clinical experience has taught me is that frail elderly patients with chronic diseases can develop unpredictable symptoms and complications and deteriorate very quickly. I don’t know how much a risk prediction tool will help in those situations.’* (Hospital consultant geriatrician, 7 years’ experience)

A community GP emphasised that decisions regarding care de-escalation, deprescribing, and personalised care planning should be informed by clinically meaningful tools rather than existing ‘blunt instruments’:

*‘When I ran electronic frailty index (eFI) on EMIS* [IT system]*, it told me fifty-one per cent of my patients had either mild, moderate, or severe frailty, and of those, forty were severely frail. I applied the Fried Frailty Phenotype Criteria and Rockwood to those forty patients and eight were probably severely frail. It showed me that eFI was heavily weighted towards MLTC and age, but it wasn't nuanced enough to pick up MLTC managed well.’* (Community GP/Clinical Lead, 22 years’ experience)

It was perceived by professionals that GPs were better placed to use prediction tools to decide on the most appropriate course of action, basing this on clinical judgement, *‘because some patients don’t need to come to hospital and some patients should never come to hospital’* (Hospital consultant geriatrician, 7 years’ experience):

*‘It would be something that I would expect primary care teams to be doing as part of their role, you know? It’s not like a tick -box exercise. It’s just part and parcel of whatever assessment they are doing.’* (Social worker, 4 years’ experience*)*

*‘I think GPs are best placed to identify high-risk patients, but GPs may not feel they're best placed. Maybe it’s geriatricians, and maybe it’s actually a different kind of physician ... I think there will always be a need for a clinician’s oversight of the patient, even though you may have risk prediction tools.’* (Hospital consultant geriatrician, 19 years’ experience)

Even if risk prediction tools were helpful to professionals, some community professionals were uncertain about how best to communicate risk and its impact on patients’ behaviour:

*‘The challenge for me is really the complexity of the messaging to patients, the statistics, and data used and what that means for me and them as individuals and the effects on behaviour changes.’* (Consultant physiotherapist, 25 years’ experience)

Although some patients and carers were supportive of healthcare professionals *‘to use all the tools at their disposal to make correct clinical judgments as long as it’s not just a tick-box exercise where they put two and two together and get 54’* (Focus group 2, male patient), others were more critical. They expressed concerns about the potential harms of the use of risk prediction tools in health care, citing that ‘life and health are a lottery’, and no one can determine the risks of future accidents:

*‘Well, they* [professionals] *shouldn’t be trying to prevent anything because they are using a tool that nobody knows how accurate it is. They could be putting extra strain and worry, giving you mental health problems that you don’t have. It can cause more problems than it can fix.’* (Focus group 2, male patient)

Some patients and carers emphasised the value of trusted professionals communicating health risks, ensuring a sense of control and clear guidance on subsequent actions is provided:

‘*If I got flagged up for something wrong, I would like to know. I appreciate it would be better if it was from someone you knew and you trusted, and they put it in a way that you had some autonomy yourself and you were treated like an individual.*’ (Focus group 6, female patient)

### Workload implications of risk prediction

GPs highlighted the importance of consistent approaches to identifying patients at risk of adverse events and standardising care pathways for patients with MLTC and frailty across boundaries and care transitions. However, they identified capacity issues in adhering to existing guidance and in implementing tools lacking clear instructions on how to respond to prediction recommendations:

*‘Prediction tools are useful only if you're going to offer some kind of help on how to manage that information. Because if you present the currently failing GP practices with information and they don’t have any capacity to deal with that and they’ve got a lot of people who are actually seeking help, then it just is another burden.’* (Community GP, 8 years’ experience)

One service manager stressed that the consistent application of prediction tools and care pathways across the whole system is challenged by general practices operating under an independent contractor model, requiring ongoing negotiations about new work:

*‘It’s different with GPs in that there’s quite a lot done by negotiation and through enhanced contracts as well as the General Medical Services contract. When we speak to GPs, nobody thinks this is a bad idea, but it’s about where that fits in with their pricing scale and having the time and ability to apply it properly.’* (Older people’s community service manager, 5 years’ experience)

Patients and carers noted limited value in risk prediction without healthcare professionals having a detailed discussion of risk implications with them. They highlighted the importance of addressing personal fears when communicating risks and questioned whether doctors would engage in meaningful conversations about these fears or simply present statistics:

*‘If the risk is calculated, would it be discussed with you, or would the professionals say: “Oh here, look at all these statistics?” They should discuss these risks with you, what you should do and also what frightens you most about it. Would they do that? And who would monitor my progress?’* (Focus group 4, female patient)

Patients emphasised that risk prediction should be an interactive and ‘ongoing back and forth’ process rather than a one-time event. They noted the need for personalised discussions that account for individual experiences and concerns with the care plans and treatment they offer:

*‘It needs to be interactive because sometimes they say, “Well, this is what we recommend you take based on your scores.” Well, I can’t take that medication any more; what do we do instead? … It’s not that I don’t want the medication; it’s just I’ve had so many problems with it, and then every time I try a new medication, it makes me very nervous.’* (Focus group 5, female carer/patient)

### Reconfiguration of risk prediction tools

Healthcare professionals emphasised the importance of developing new risk prediction tools that have high accuracy in identifying at-risk patients. To enhance their effectiveness, the tools should ideally be integrated with clinical decision support tools and/or lead to tailored interventions, and incentives alongside resources for upskilling should be provided to improve uptake:

*‘One of the things that GPs really respond to is big data. Also, what does the introduction of a new tool mean for people? Are we preventing a crisis by intervening early enough?’* (Social care service manager, 2 years’ experience)

*‘New tools have to be coupled with some kind of intervention that’s easy to access and doesn’t involve lots of paperwork because we can't tolerate extra work … So, you identify twenty people at risk of admission, and then there is a package of effective interventions you are trained to implement.’* (Community GP, 6 months’ experience)

New tools requiring additional steps to access or ones hidden within apps were perceived as unlikely to be adopted. Ideally, tools should be embedded in the existing care pathway, providing clear and user-friendly indicators. Clinicians valued tools that generate scores to support clinical decisions, but expressed concerns over integrated alert warnings. ‘Alert fatigue’ caused by an excess of notifications was cited as a major limitation, affecting the uptake of such tools in routine practice:

*‘There’s a careful balance to be struck. When you prescribe a medication and if patients are over a certain age, the box will flash with advice: “Be aware that this is more likely to cause a fall for the elderly, or this is a STOPP medication.” .But because that crops up every time you prescribe something, you often ignore it.’* (Out-of-hours GP, 1 year’s experience)

The need for risk prediction for rapid functional decline and falls (both in and outside of inpatient settings), delirium, and sepsis was identified. Additionally, tools that predict adverse drug events leading to acute admission to hospital, which accounted for not only drug–drug interactions but also patient psychosocial characteristics, clinical symptoms and test results, were highlighted:

*‘The biggest and most expensive risk is falls and fractures, and we struggle a lot in terms of accurately identifying and managing these.’* (Hospital consultant geriatrician, 19 years’ experience)

Healthcare providers discussed the potential of artificial intelligence (AI) and electronic health records in developing new risk prediction tools, emphasising the importance of creating algorithms that also focus on outcomes meaningful to patients, for example, quality of life and treatment burden:

*‘Patient work is the tool that would be helpful. Every time you add a new diagnosis and prescription, there are potential implications for the amount of work patients will do to negotiate our systems … and polypharmacy problems caused.’* (Clinical lead in general practice, 22 years’ experience)

Patients and carers expressed scepticism about the need for new prediction tools, arguing that current NHS resources would be better allocated to improving access to care and care quality. If new tools were to be developed, their accuracy must be ensured through the use of comprehensive medical histories. Patients noted that some older individuals might be less inclined to respond to scores generated by prediction tools, as they may feel less concerned about future risks given their age:

*‘They* [professionals] *are wasting all their money in developing these predictive tools that would be better spent elsewhere … Our biggest thing is we can’t get an appointment … If they develop tools predicting life-threatening conditions, I can see the point. But if you are old and you have got this far, you are quite happy to continue the way you are.’* (Focus group 2, male patient)

## Discussion

### Summary

Our findings underscore the importance of establishing legitimacy for risk prediction tools among professionals, patients, and carers. Professionals raised concerns about the clinical utility of existing prediction tools for patients with MLTC and frailty, suggesting current algorithms often lack the reliability needed to account for complex and unpredictable patient health trajectories. Operational demands of implementing existing tools and the lack of seamless integration into clinical workflows were seen to increase workload and contribute to ‘alert fatigue’, limiting adoption in practice.

They further noted that new tools need to be demonstrably accurate in patients with MLTC, target a range of outcomes meaningful to patients, and incorporate actionable guidance that considers patient preferences. AI and routinely collected data were viewed as valuable for improving predictive accuracy and enabling real-time tool use; however, their effective implementation will depend on robust IT infrastructure, adequate training, and ongoing clinical oversight — ‘human-in-the-loop’.

Similar to professionals, patients/carers expressed uncertainty about the accuracy of prediction tools, alongside concerns that their use in older age might be less useful and provoke unnecessary anxiety or undermine autonomy. They further emphasised the importance of personalised risk communication over generic statistics to support informed decision making.

Using normalisation process theory^29^ as an interpretive lens, our findings align with the theory’s core constructs. Questions of legitimacy (clinical credibility, practical acceptability, usefulness, and relevance) correspond with the constructs of coherence (understanding the purpose/value of the tool) and cognitive participation (willingness to invest in and promote the tool), reflecting the need for professionals to make sense of and commit to using new tools in practice. Perceived barriers to seamless integration into clinical workflows were found, including additional workload, lack of interoperability or alert fatigue, and challenges related to collective action, limiting adoption into everyday practice.

Furthermore, the call for robust validation, personalised risk communication, meaningful outcome targeting, and actionable outputs aligns with reflexive monitoring (ongoing evaluation of the tool), where professionals, patients, and carers assess added value against associated burden within the local context. Although AI-driven tools were viewed as potentially transformative, their successful implementation hinges on investment in IT infrastructure and workforce training. These insights underscore that normalisation of risk prediction tools depends not only on technical performance but also on how well they are embedded within the social and operational realities of routine care for patients with MLTC.

### Strengths and limitations

The study’s strengths include the targeted recruitment of a diverse group of health and social care professionals, as well as patients with MLTC and carers. However, recruitment was restricted to a single large Scottish health board, which may limit the transferability of findings to other geographical areas or health systems that operate under different organisational structures, policies, or resource constraints. Our patient/carer sample was gender and geographically balanced; however, most of the participants were older adults from White ethnic backgrounds. As a result, the findings may not fully reflect the perspectives and priorities of working-age people with MLTC and those from ethnic minority backgrounds, which could be usefully addressed in future research.

### Comparison with existing literature

Previous research suggests that professionals value risk stratification tools for their structured, evidence-based approach to risk assessment. However, prediction tools' performance is commonly evaluated in the whole population, with limited evidence on their accuracy and reliability in people with MLTC and frailty. Recent studies show that risk prediction tools perform suboptimally in patients with MLTC defined by the Charlson Comorbidity Index.^30,31^ For instance, QFracture-2012 systematically underpredicted fracture risk in the whole population, but overpredicted the risk in those with MLTC.^30^ Previous research suggests that including clinical judgement-based metrics such as clinical frailty scale and functional status can enhance risk predictions in patients with MLTC, who often experience health declines that are not captured by disease-focused indices.^32^ Similar to us, previous research has also shown that patients’ interpretations of risk communication are shaped by social dynamics and everyday life concerns, with anxiety and self-absorption viewed as common experiences if discussions lack a personalised approach.^33^ Therefore, it was suggested that situating risk communication within patient realities and social contexts can align risk discussions with patient values and improve engagement.

### Implications for research and practice

There is a need to develop risk prediction tools derived from routinely collected data that better reflect the characteristics and complexity of patients with MLTC and frailty. These tools should be seamlessly integrated into existing IT systems and embedded within routine clinical workflows to optimise accessibility and uptake,^10,11^ which can be achieved by investment in user-centred interfaces and interoperability with IT systems.

New tools should aim to reduce workload for already stretched clinicians by deprioritising individuals at low risk who currently trigger unnecessary reviews because of the limitations in existing systems to tailor responses to prediction appropriately. Further, for risk prediction tools to add value to clinical decision making, outputs must be interpretable, clinically meaningful, and presented in ways that support clinical judgement.

Capacity to respond to prediction recommendations in a person-centred way was noted as important by patients/carers.^34^ Clinicians, therefore, would benefit from structured, ongoing training in effectively interpreting, applying, and communicating risk to patients. Effective risk communication requires an approach that balances transparency about health risks with sensitivity to avoid inducing fear or anxiety. Motivational interviewing is increasingly recognised as an effective method for such communication, as it focuses on collaboration, empathy, and patient empowerment, making it suitable for patients with MLTC.^35^ It encourages practitioners to frame information in a way that resonates with patients’ personal goals and values, which helps them internalise the information more effectively.^36^

Prediction models should be developed and validated within cohorts of patients with MLTC using clinically meaningful outcomes and advanced analytical methods capable of capturing non-linear and dynamic health trajectories. Comparative studies assessing these tools against current standards are essential to confirm their added value in risk identification and decision making.^10^

To ensure successful adoption, co-production approaches with clinicians, patients, and other stakeholders will provide important insights into factors influencing tool acceptance and optimal implementation practices. These approaches will also inform the development of tailored risk communication strategies, especially for patients with limited health literacy or elevated anxiety, promoting better patient engagement and outcomes.

Further research will benefit from examining whether and how the acceptability of specific risk prediction tools varies between professionals and patients, for example, because of variation in which outcomes are most valued.^37,38^ Equally important is addressing implementation through integration into clinical workflows, supported by appropriate IT infrastructure and training, with ongoing clinical oversight.
